# E-Skin Development and Prototyping via Soft Tooling and Composites with Silicone Rubber and Carbon Nanotubes

**DOI:** 10.3390/ma15010256

**Published:** 2021-12-30

**Authors:** Josué García-Ávila, Ciro A. Rodríguez, Adriana Vargas-Martínez, Erick Ramírez-Cedillo, J. Israel Martínez-López

**Affiliations:** 1 Tecnologico de Monterrey, Escuela de Ingeniería y Ciencias, Monterrey 64849, Mexico; garcia.josue@tec.mx (J.G.-Á.); ciro.rodriguez@tec.mx (C.A.R.); adriana.vargas.mtz@tec.mx (A.V.-M.); 2 Laboratorio Nacional de Manufactura Aditiva y Digital (MADiT), Apodaca 66629, Mexico; 3 3D FACTORY MX, Ramon Treviño 1109, Monterrey 64580, Mexico; 4 Centro de Investigación Numericalc, 5 de mayo 912 Oriente, Monterrey 64000, Mexico

**Keywords:** additive manufacturing, electronic skin, Low-Force Stereolithography, room-temperature-vulcanizing, RTV, single-walled carbon nanotubes, soft tooling, stereolithography, SWCNTs

## Abstract

The strategy of embedding conductive materials on polymeric matrices has produced functional and wearable artificial electronic skin prototypes capable of transduction signals, such as pressure, force, humidity, or temperature. However, these prototypes are expensive and cover small areas. This study proposes a more affordable manufacturing strategy for manufacturing conductive layers with 6 × 6 matrix micropatterns of RTV-2 silicone rubber and Single-Walled Carbon Nanotubes (SWCNT). A novel mold with two cavities and two different micropatterns was designed and tested as a proof-of-concept using Low-Force Stereolithography-based additive manufacturing (AM). The effect SWCNT concentrations (3 wt.%, 4 wt.%, and 5 wt.%) on the mechanical properties were characterized by quasi-static axial deformation tests, which allowed them to stretch up to ~160%. The elastomeric soft material’s hysteresis energy (Mullin’s effect) was fitted using the Ogden–Roxburgh model and the Nelder–Mead algorithm. The assessment showed that the resulting multilayer material exhibits high flexibility and high conductivity (surface resistivity ~7.97 × 10^4^ Ω/sq) and that robust soft tooling can be used for other devices.

## 1. Introduction

**Figure 1:**
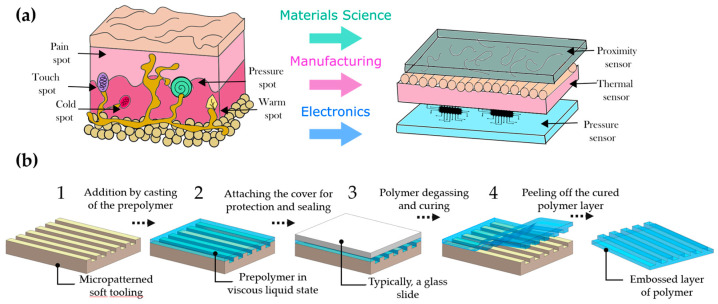
(**a**) Structure and sensing mechanisms of artificial electronic skin (**right**) and biological skin (**left**); (**b**) schematic illustration of the fabrication process of casting layers using soft tooling; (**1**) the first step is to clean the mold, (**2**) next, in the second step, the material is cast into the mold; (**3**) after coating and sealing via a protective substrate, the third step consists of degassing using some vacuum chambers and some negative pressure sources; finally, (**4**) curing is performed in a convection oven or other heat source prior removal.

The artificial recreation of tactile sensing is vital for developing more natural interaction between robots and the environment. Synthetic recreation in this sense could enhance remote online interactions and ultimately be part of a fully regenerative medicine scheme for limbs or other body parts. The development of electronic artificial skin or e-skin is a complex problem that deals with diverse sciences and disciplines, including electronics (soft robotics, wearables, haptic actuators, and neuroprosthesis control), bioengineering and materials sciences (tissue regeneration, personalized medicine, biosensors), and manufacturing (polymer solution casting, inkjet printing) [1,2], as can be seen in Figure 1a. New materials and manufacturing procedures have enabled the production of larger and more sensitive surface areas [3]. The structure of human skin has inspired researchers to recreate the functionality of these tissues using multilayered material structures. Artificially constructed tissues sense environmental conditions, such as humidity, pressure, or temperature [4,5]. Furthermore, conditions such as pH, blood oxygen saturation, heart rate, blood pressure, and muscular or neuronal electrical activity have been monitored using artificial skin [6,7].

There is a wide variety of transducers for sensing contact based on electromechanical, photoelectric, or electrochemical phenomena. These signals are generated in arrays of stacked materials (substrates and electrodes) regardless of the source. The substrates are mechanical foundations that support electrode layers that feature conductive properties that allow their sensing function. For instance, the electrodes are typically arranged in a 2D row–column matrix pattern creating overlapping intersections, which allows the generation of mutual capacitance between each pair of receivers (i.e., column) and transmitters (i.e., row); the capacitance sensitivity and detection range is intimately linked to the geometry of these electrodes.

Some of the polymers used to manufacture the substrates are based on polydimethylsiloxane (PDMS) [8], polyethylene terephthalate (PET), butyl rubber (IIR.), room-temperature-vulcanized silicone-rubbers (RTV) [9], and styrene-butadiene-styrene rubber (SBS) [10]. Electrodes are typically made from nanocomposites with conductive nanoparticles [11]. For example, Single-Walled Carbon Nanotubes (SWCNTs) embedded in PDMS thin films exhibit high sensitivity, fast response times, and excellent stability [12]. Research about the synthesis of conductive R.T.V. silicone rubber composites layers with cross-linking of conductive fillers is limited to manufacturing processes such as Aerosol-Jet-Printing [13], or a more straightforward process to cast film samples [9,14].

Additive manufacturing (AM) has played an essential role in manufacturing freeform flexible structures for biomedical devices. However, some scenarios are not feasible considering the limitations on the availability of materials or difficulties for post-processing the sample [15], especially for two-component silicones, such as the RTV-2 [16]. Indirect development using soft tooling (molds) produced using additive manufacturing has emerged as a cost-effective, scalable, customizable manufacturing alternative [17,18] for such cases. This approach reduces manufacturing times, can be adapted to low-scale production scale, and has proven helpful in early production or new product prototypes [19]. Modern additive manufacturing processes, such as stereolithography (SLA), allow the production of soft tools with high precision and dimensional resolutions [20]. Although these are made from polymers, new ceramic and metallic metal printing advances are emerging. These options can improve curing times or enable new features. The flexibility of manufacturing devices with three-dimensional features can enhance the throughput for the early steps of the product life cycle. For example, different patterning cavities enable the generation of different device elements with fewer manufacturing steps. Developing and testing multi-cavity and multi-purpose soft tooling design methodologies can bolster artificial electronic skin manufacturing processes.

In recent years, soft tooling has evolved to develop configurations with more complex and intricate mold geometries. Standard geometries encountered are conical pillars (needles), rectangular or square prisms, cylindrical pillars, or tetrahedral pillars [21,22]. Geometries that do not feature a taper experience adhesion problems that cause damage during peeling off. Some recommended techniques include the use of draft angles, rounded edges, and reducing the surface roughness of the mold [23]. A typical manufacturing process of artificial electronic skin is divided into four simple consecutive steps (see Figure 1b). However, the inherent difficulties associated with additive manufacturing using vat photopolymerization include processing times that are typically long and result in diminished thermo-mechanical properties [24]. Hence, soft tooling requires that the mold withstand the stress produced by heat and forces during manufacturing. Furthermore, while S.L.A. printing resolution excels among AM technologies, the build volume (working space of the devices) is smaller than that of more traditional technologies, such as Fused Deposition Modeling (FDM) [25].

There are diverse approaches to characterize the effect of carbon nanotube polymerization kinetics, including the presence and concentration of catalyzers [26], the piezoelectric performance [27], and the heat transfer response [28]. Reviews of CNTs published recently demonstrate the vast scope of the topic [29,30].

Room-temperature-vulcanizing silicone (RTV-2) is a low-molecular-weight dimethyl polysiloxane-based rubber that can be cured without an external energy heat source and is widely used in sealing applications in the automotive industry, medical prosthetics, and electronic encapsulation. The development of e-skin based on RTV materials has been reported previously, but the manufacturing conditions have not been tested thoroughly.

One of the most critical steps during the production of silicone-based products is degassing, since it eliminates all the bubbles formed during the mixing stage. However, bubbles can appear during the casting of the material in open molds; some have explored the technique of centrifugal casting in closed molds [31] or the development of low-cost automatic vacuum casting systems [32]. In the case of nanocomposites based on RTV silicones, the temperature becomes another relevant variable because it accelerates the curing process and reduces the time available for the vacuum degassing process. However, the nanocomposite viscosity increases because of premature curing and the inclusion of SWCNTs. According to Vakili-Nezhaad et al. [33] the viscosity is increased up to 32.94% at a weight fraction of 0.2%. It is essential to characterize the effect of the conductive matrix because the mechanical and electrical properties of the composite depend on their ratio. Another element to consider is the methodology for mixing the CNT with the matrix. Kundalwal and Rathi recently studied the effects of ultrasonic processing and magnetic stirrers for a dual mixing strategy for multiwalled carbon nanotoubes (MWCNTs) [34].

In work reported previously, we showed that Low-Force Stereolithography (SLA-LF), a variation that employs a flexible V.A.T. tank to reduce the forces exerted on parts during the manufacturing process, could produce polydimethylsiloxane (PDMS) casting molds [18]. The aim of this study is threefold: first, we want to gain new insight regarding the manufacturability of molds for nanocomposites; second, we would like to assess the effect of the organic filler on the mechanical properties and conductivity properties for a nanocomposite; and finally, we would like to generate a framework for future robust design of molds. This study is of relevance because it addresses limitations from the manufacturing point of view.

To fulfill these objectives, we first conducted an experimental study of additive manufacturing with an initial mold design (an assay on the fabrication of layer RTV-2 and SWCNT-based nanocomposite structures) and modeled their mechanical properties. Next, with the insights provided by the study and a reexamination of existing research, we proposed design guidelines for the AM-based soft tooling of nanocomposite-based devices.

The methodology followed in this study provides insights and technical considerations that can be useful for the design of an alternative cost-effective rapid manufacturing methodology. Multi-cavity and multi-purpose soft tooling could be a resource to accelerate the manufacturing and sample testing processes.

## 2. Materials and Methods

### 2.1. Artificial Skin Design

**Figure 2:**
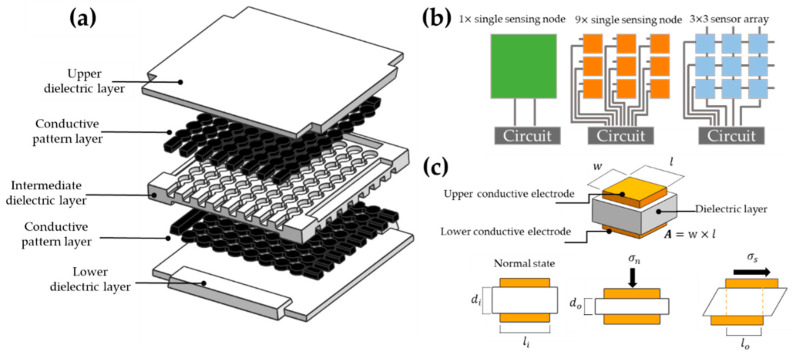
(**a**) Schematic illustration of the proposed all-elastomeric skin-like pressure sensor array; (**b**) conceptual topologies diagrams of array sensor; (**c**) principle of operation to measure normal and shear stress.

The flexible sensor proposed here is a laminated structure with an active sensing area of 25.4 mm × 25.4 mm (cavity size) conformed by 64 individual electrodes (see Figure 2a). The sensing can be achieved using the nanocomposite material SWCNTs/RTV-2 to create a highly conductive array. While the outer and intermediate layers are made of elastomeric material, an external multiplexed data acquisition circuit is connected to each row (*i*), and column (*j*) of the conductive layer array (see Figure 2b). The topology of the array determines the spatial pressure resolution of the e-skin. The large deformation and flexibility of the nanocomposite makes it possible to vary the area of overlapped electrodes in the stacked layers. A change in capacitance due to deformation by an external force can be detected using the circuitry. Furthermore, the ability of these sensors to measure shear stress ($\sigma_{s}$) in addition to normal stress ($\sigma_{n}$) is a significant advantage. Although capacitive sensors require sophisticated electronic components, they have been found to provide greater sensitivity and flexibility, less dependence on temperature, more robust structures, low power consumption, better frequency response, and superior dynamic range compared with piezoresistive devices [35].

### 2.2. Different Formultions with Vaying Materials for Nanocomposites

ELASTOSIL^®^ P 7600 and ELASTOSIL 7683 RTV polymers (Wacker Chemie AG, Adrian, MI, USA) were tested on this work. The properties reported by the manufacturer are shown in Table 1.

Nanofiller contents (%) were prepared (Sample I, Sample II, and Sample III) (see Table 2). The RTV produced by the manufacturer (Wacker Chemie AG, Adrian, MI, USA) comprised parts A and B and were mixed in a 1:1 ratio. To form the nanocomposite, carbon SWCNTs Tuball™ Matrix 601 (>70% carbon nanotube content, diameter 1.6 ± 0.4 nm, and G/D >100, Columbus, OH, USA) was used.

### 2.3. Nanocomposite and Substrate Fabrication

**Figure 3:**
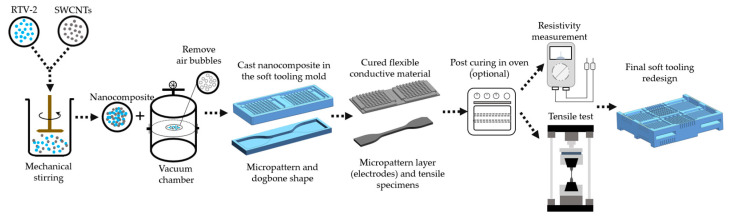
Schematic illustration of the assessment process for nanocomposites RTV-2/SWCNTs.

The process flow for the fabrication of the SWCNTs/RTV-2 nanocomposite for tensile specimens and micropatterns layers is shown in Figure 3. Part A and Tuball™ Matrix 601 were pre-mixed using a mechanical stirrer at 1000 revolutions per minute (RPM) for 15 min. Next, the curing agent (RTV-2 part B) was added and stirred (15 min and 1000 RPM). Finally, the sample was moved into a vacuum chamber for deaeration using a vacuum oil pump with a 5 ft^3^m^−1^ displacement speed (25 µmHg for 5 min). Through visual inspection, care was taken to assure that bubbles were not present in the specimens.

### 2.4. Microelectrode Fabrication and Assessment

**Figure 4:**
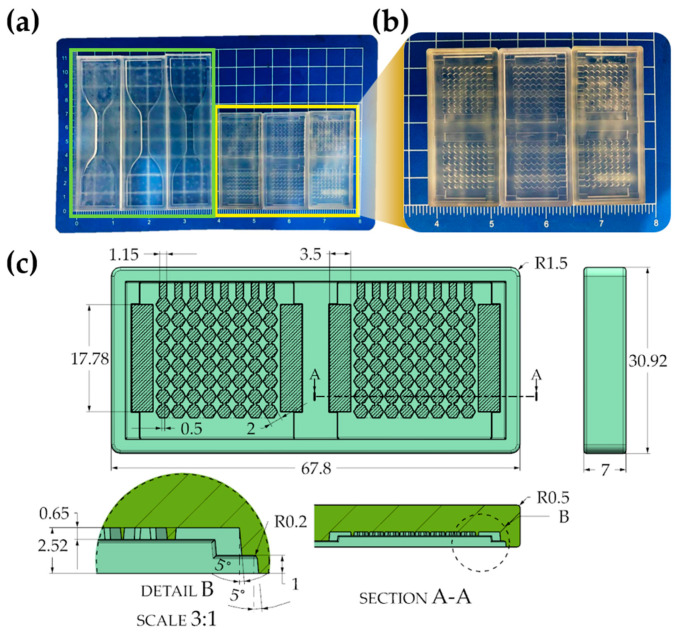
(**a**) Photos of three molds for tensile specimens (green box) and three initial two-cavity molds (yellow box) printed by Low-Force Stereolithography (x- and y-units show dimensions in inches and centimeters respectively); (**b**) detail of two molds with hexagonal micropattern and one mold with circular micropattern (40% modified contrast); (**c**) overall dimensions of the hexagonal pattern geometry and soft tooling cavities (all dimensions in mm).

All the molds (including the tension specimens; Figure 4a, green box) were designed using Siemens NX 12. On the other hand, the molds with microgeometry presented in Figure 4b were first manufactured with two cavities with 2 mm circular micropatterns and the third mold with 2 mm hexagonal microgeometry; their relevant geometric details can be seen in Figure 4c. The overall sizes of these two-cavity soft tooling and specimen tensile molds were 67.8 mm × 30.92 mm × 7 mm and 107 mm × 31 mm × 5.5 mm, respectively. The molds were printed with their larger side parallel to the print bed.

### 2.5. Soft Tooling Manufacturing Using Stereolithography-Based Additive Manufacturing

A benchtop SLA-LF Form 3 additive manufacturing equipment (Formlabs, Somerville, OH, USA) was used, employing a 50 µm for the high-temperature FLTHAM02 for manufacturing all the molds in this paper. The samples were cured using the provider’s recommended settings for post-processing the sample accordingly (120 min at 160 °C) on a hot plate.

### 2.6. Mechanical Characterization

To test the mechanical properties of the materials and compare them for different nanocomposite compositions, tensile tests were conducted.

The tensile tests were performed based on a standardized method considering a Type IA specimen with an overall length of 100 mm and a 3 mm thickness, which met all the specifications listed standard ASTM D412-16 (2021) [36]. A universal testing machine (3365, INSTRON, Norwood, MA, USA) equipped with a 50 kN load cell was used, considering a crosshead speed of 500 mm/min. Three specimens of each type of continuous pure material and assessed composition (Sample I, Sample II, and Sample III) were loaded axially and monotonically at a speed deformation of 0.3 mm/s until complete failure. Next, loading-unloading uniaxial cyclic tests were performed at three different maximum strain levels (ε=4, 3, 2) for 10 continuous cycles. After the first couple of load cycles, the material stress–stretch response become repeatable [37].

The experimental results of the loading–unloading tests were fitted via inverse analysis with predicted data curves based on the Ogden–Roxburgh model using the Nelde–Mead optimization algorithm, as suggested in [38] and described in Section 2.4. The fitting step was intended to optimize the Ogden–Roxburgh model. The coefficient of determination R^2^ was calculated for the predicted results and compared to the experimental data for every iteration step. These values are then summarized as the descriptor for the objective minimization function for the next iteration step of a Nelder–Mead optimization loop [39].

To monitor the temperature during the mixing of the elements of the nanocomposite parts, a 640 × 480 pixel Flir ONE thermal imaging camera (Teledyne FLIR LLC, Wilsonville, OR, USA) was used.

A visual inspection of the features of the microelectrodes was performed using an S.Z.M. AmScope stereoscopic microscope (United Scope L.L.C., Irvine, CA, USA). The quality inspection of the defective single electrodes of each 8 × 8 matrix array was performed via a nonparametric Kruskal–Wallis test with multiple pairwise comparisons between groups. Considering the variability of possible defects between different concentrations of SWCNTs, distribution symmetry was not assumed, and the median was calculated instead of the mean. Pairwise differences between treatments were assessed using the Dunn–Bonferroni method, and the significance level α was 5%. All the statistical analyses were performed in SPSS^®^ Statistics version 28.0.1.

To corroborate the surface electrical conductivity, samples of 15 cm × 15 cm and 3 mm thickness were prepared with the nanocomposite types I, II, and III. These samples were screened on an analog DESCO model 19,784 surface resistance meter (Desco Industries Inc., Chino, CA, USA). This is a portable device that produces a signal if a resistance higher than 10^5^ Ω is detected. The methodology employed to measure the resistance was point-to-point (RTT, or Resistance Top to Top).

Surface resistivity $\rho_{s}$ is a physical property of a material and the surface resistance $\;R_{s}$ (also known as sheet resistance) depends on the material and the geometry of the electrodes (probes) used in the measurement. To differentiate between them, $\rho_{s}$ is often expressed in Ω/sq. The relationship between both characteristics according to standard ASTM D257 [40] for circular electrodes is:

(1) $$\rho_{s}=\frac{\pi \times D\times R_{s}}{g}$$

where g is the gap between the electrodes and *D* is the diameter of the electrodes.

### 2.7. Hyperelastic Model Based on Mullins Effect

This strain-softening phenomenon (also called the Mullins effect) presented in the elastomeric matrix was predicted using the Ogden–Roxburgh hyperelastic constitutive model [41], which defines isotropic incompressible materials’ strain energy function $W(\lambda_{i},\eta )$ under quasi-static loading. Several works have successfully used this phenomenological model to obtain a predictive model for high-strain elastomeric soft materials [42,43]. To compare the model with our available experimental results, we considered the simple uniaxial loading case, in which the principal stretches ratio are $\lambda_{1}=\lambda$, $\lambda_{2}=\lambda_{3}=\lambda^{-1/2}$; we also write $\sigma_{1}=\sigma$, $\sigma_{2}=\sigma_{3}=0$. Next, the adapted strain energy function can be expressed as:

(2) $$W\left(\lambda ,\eta \right)=\eta W_{0}\left(\lambda \right)+\phi \left(\eta \right)$$

(3) $$W_{0}\left(\lambda \right)=\overset{N=3}{\underset{i=3}{\sum }}\frac{\mu_{i}}{\alpha_{i}}\left(\lambda^{\alpha_{i}}+2\lambda^{-\frac{1}{2}\alpha_{i}}-3\right)$$

where $W_{0}\left(\lambda \right)$ is the original stress-energy function based on the classical Ogden nonlinear elastic model (*N* = 3), and ϕ(η) is a smooth energy damage function that depends on the scalar damage variable η evolving towards softening, as expressed using a Gaussian error function:

(4) $$\eta =1-\frac{1}{r}\mathrm{erf}\left[\frac{W_{\max}-W_{0}}{m+\beta W_{\max}}\right]\;$$

where r, m,and β are material-dependent dimensionless parameters, r (always r≥1) is a measure of the extent of the damage relative to the virgin stress-strain behavior, m (always m≥0) defines the dependence of the damage phenomenon on the extent of deformation and β (always β≥0) specifies the slope of the softening curve compared to the initial loading curve, and $W_{\max}$ is the maximum strain energy potential function over the range deformation history. In our case, the calibration process for a three-order Ogden–Roxburgh (N = 3) prediction ensured an adequate match between the predicted equation and the stress response of the experimental data. As in the polynomial form approach, there is no limitation on the parameter *N*. A higher value may provide a better fit for the exact predicted solution. However, on the other hand, it may cause numerical difficulty in computational consumption when fitting the material constants and requires enough data to cover the entire deformation range of interest. Therefore, a value of N>3 is not usually recommended. This latter procedure was performed using parameter-extraction software, namely MCalibration^®^ 6.5.1 by PolymerFEM [44] with integrated internal Ansys and Abaqus solvers.

## 3. Results and Discussion

### 3.1. Nanocomposite and Substrate Fabrication

#### 3.1.1. Challenges Associated with the Processing of SWCNT Nanocomposites

Single-Wall Carbon Nanotubes (SWCNTs) are highly effective additives for improving polymer-based material conductivity. However, the attractive Van der Waals interactions between SWCNTs result in them agglomerating. Ideally, we must disperse separate nanotubes into the base material to achieve optimum strengthening and electrical conductivity. The Tuball™ Matrix highly concentrated masterbatch (SWCNTs homogeneously dispersed in polydimethylsiloxane PDMS) used in this work facilitated the well-dispersed of nanotubes loading fractions into the RTV-2 matrix in our sample with high concentrations (up to 5 wt.%). In this context, Pötschke et al. [45] reported that nanocomposites containing <2 wt.% nanotubes exhibited Newtonian behavior, quite similar to that of the unfilled matrix. This SWCNT-based masterbatch also avoided a widespread problem at the time of scalability of the product, especially the level of percolation threshold required to achieve adequate electrical conductivity in the nanocomposite with low concentration (2 wt.%).

**Figure 5:**
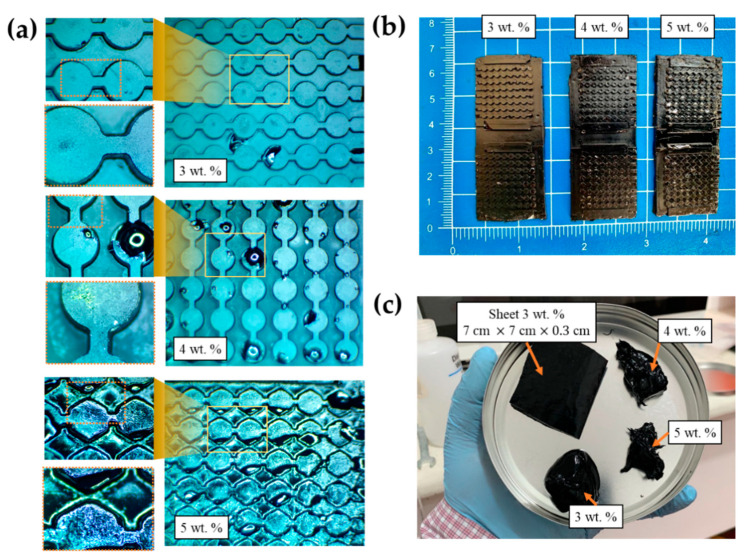
(**a**) Images from an optical microscope to inspect the definition of edges and defects in the patterns for different concentrations of nanotubes; (**b**) results obtained from peeling off without transverse detachment of the samples; (**c**) consistency of the viscous mixtures for different concentration of nanotubes.

However, the increase in viscosity of the masterbatch can limit the diffusion and sedimentation of SWCNTs by restricting the Brownian motion. According to the manufacturer, the best compatibility using the dilution process via a high-speed overhead stirrer is achieved with R.T.V. with low-viscosity (up to 50,000 mPa·s) and not more than 2 wt.%. Experimental data show that these results can be achieved even at higher concentrations without a pre-dilution process. Consequently, the cross-linking and viscosity are greatly accelerated with increasing temperatures, so the heat generated during mixing must be strictly controlled. This viscosity condition can be make it challenging to process nanocomposite during the final casting in soft-tooling and can cause high porosity levels, as shown in Figure 5a. However, RTV-2 features low surface tension, which also allows an excellent molding pattern replication.

On the other hand, the pot life of the elastomers used in both cases, which usually indicates the maximum period during which the catalyzed mixture of part A and part B is processable (or during which the initial viscosity doubles its value), did not exceed 1 h. Nevertheless, the presence of exothermic enthalpy of PDMS from the masterbatch caused its nanocomposite to reduce the pot life in the samples with 5 wt.% loading fractions. Figure 5b shows the peel-off substrates after the supplier’s pot life and the curing phenomenon’s alteration.

#### 3.1.2. Defect Inspection of Nanocomposite Micropatterned Layers

**Figure 6:**
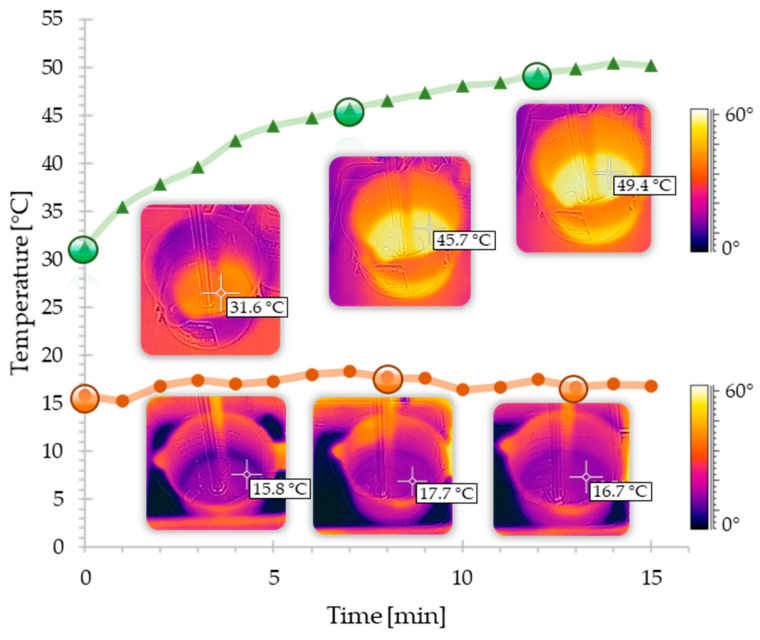
Temperature monitoring over 15 min in mixing processes assisted with and without a reverse bain-marie; the manufacturer’s recommended temperature is <20 °C.

The concentration of SWCNTs and temperature can significantly modify the rheological properties of SWCNT OCSiAl masterbatch concentrations that exceed 1 wt.% [46]. For this paper, the concentrations of SWCNTs could cause air-trapped defects in the soft tooling electrodes shapes due to the high viscosity; however, it is feasible to process the samples in sheet form without these defects occurring, as can be seen in Figure 5c. While we were able to achieve a free-solvent homogeneous nanocomposite mixture, the mechanical work of the stirrer increased the temperatures by about 50 °C. One way to mitigate the heat transfer was to employ a cold-water bath. Figure 6 shows that employing this method made it possible to maintain a constant temperature, around 17 °C.

The inspection of the defective electrodes in the micropatterned layers manufactured during this study o caused by SWCNT concentrations in the three groups (k = 3) with different filler concentrations was considered entirely independent; the test is based on the null hypothesis H_0_ on the median ($\tilde{\mu }$) of the number of defective electrodes that assumes ${\tilde{\mu }}_{1}={\tilde{\mu }}_{2}={\tilde{\mu }}_{3}$ against the alternative hypothesis H_1_, where at least one median is different from the rest. The test statistic K was used, which followed a behavior comparable with the chi-square distribution ($\chi^{2}$) with k−1 degrees of freedom. According to the results shown in Table 3 for significance level (α=0.5), the null hypothesis was rejected (*p*-value < 0.05), so there was sufficient statistical evidence to rule out the possibility that the three medians were equal. The pairwise comparison between the study groups with different concentrations of SWNTs corroborates that all the medians were different for each of the study groups (Table 4).

#### 3.1.3. Surface Resistance Measurements Results

Our procedure required the use of two 5 lb cylindrical probes with a diameter *D* of 2.5′′ (6.35 cm) and a distance g of 2.5 cm, placing the nanocomposite material to be tested on an insulative surface, and performing the measurements in orthogonal directions, i.e., moving the probes to measure in a cross direction and repeating the test. This is a simple go/no-go procedure to evaluate the presence of high electrical conductivity in the nanocomposite. All the samples manufactured yielded a value of *ρ_s_*~7.97×10^4^ Ω/sq using Equation (1). Conductive materials have a surface resistivity of less than 1 × 10^5^ Ω/sq. On the other hand, the volume resistivity (also known as bulk resistivity, specific electrical resistivity, or bulk resistivity) $\rho_{v}$ (in Ω·cm) can be calculated by multiplying the surface resistance $R_{s}$ by the sample thickness (in cm): $\rho_{v}=R_{s}\times t$; in our case, $\rho_{v}\sim$2.39×10^4^ Ω·cm.

### 3.2. Uniaxial stress-strain Behavior and Mullin’s Effect

**Figure 7:**
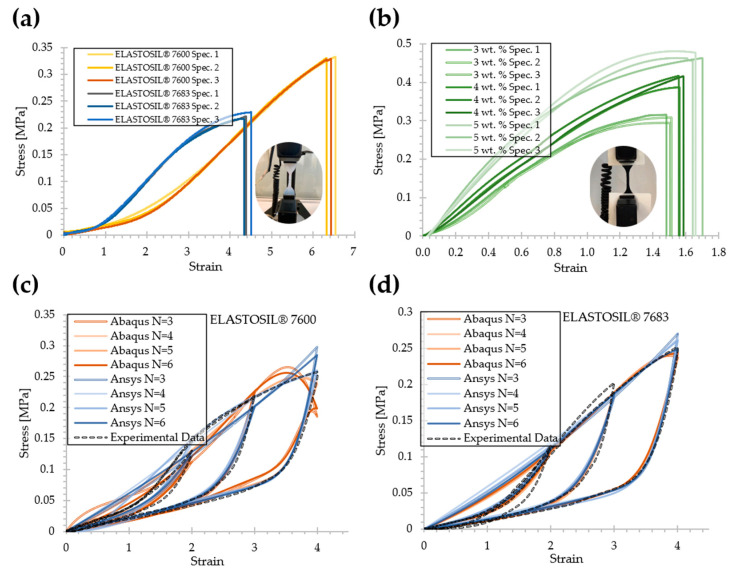
(**a**) Stress-strain curves of uniaxial tension test subjected to quasi-static axial loads of RTV-2 material and (**b**) RTV-2-based nanocomposite filled with different concentrations of SWCNTs; hysteresis curves of uniaxial stress loading and unloading on material ELASTOSIL^®^ 7600 (**c**) and 7683 (**d**).

Figure 7a shows the stress-strain experimental data records under true uniaxial stress for the pure substance. The mean elongation break was registered at $\bar{\varepsilon }\;$= 441.075% and 643.542%. The data were mostly consistent with the behavior predicted by the datasheet of 500%, 600% for ELASTOSIL^®^ 7600 and 7683 material, respectively. Figure 7b shows that with higher nanotube concentrations, the stress value for the failure point increased to 0.306 MPa (3 wt.%), 0.406 MPa (4 wt.%), and 0.465 MPa (5 wt.%). Figure 7c,d reveals that the pure elastomeric material exhibited a level of dissipated energy hysteresis, known as the Mullins effect, during cycling loading for three different stretch ratios λ={5, 4, 2}, where the strain equals the stretch minus unity (ε=λ − 1).

The Nelder–Mead optimization method and the Abaqus and Ansys solvers were used to calculate the parameters ${r,\;m,\;\beta ,\;\mu }_{i}$ and $\alpha_{i}$ (*i* = 1, 2, 3) listed in Table 5. A three-order Ogden–Roxburgh model fit with R^2^~0.87, ~0.92 of the Mullin’s effect of an ELASTOSIL^®^ 7600 and 7683, respectively. The parameters of the full Ogden-Roxburgh model summarized in Table 5 characterize the elastic strain energy of Equation (3). With this fast optimization method, fit curves were achieved before 3000 iterations (number of function evaluations); the R^2^ values were evaluated and compared in a range of *N* = {3–6} (see Table 6). The full numerical predicted curves for different systems of grade *N* are also given in Figure 7c,d.

### 3.3. Design Guidelines for AM-Based Soft Tooling

This paper’s redesign of the multi-cavity mold was approached from 10 different aspects, based on lessons learned during experimental development. The design process involved analyzing these aspects before and during the definition of the final rubout design. Although the list of the 10 aspects is not exhaustive, they tackle the sources of issues for this purpose. These specific guidelines are based on more general principles of the robust and reliability-based design optimization methodology and from the exhaustive review of lessons learned from scientific research in the field of soft tooling [47].

1. Definition of requirements and limitations. Define the overall dimensions of the multilayer structure of the artificial skin, the thicknesses of the layers independently, the quantities of layers to be assembled, the required quality standards (e.g., surface roughness), the number of cavities and cores, the volume of production, material selection, critical specifications, and geometric tolerances [48].
2. Design of the topology of the replicated structure. Define the size and depth of the conductive microchannels, the shape of the geometry engraved in the mold, the spatial orientation, distance to the cavity edges, draft angles, and rounding in intricate corners.
3. Design of the alignment system. Define the type of alignment (geometric, magnetic, mechanical, etc.), pin dowel material, locking units, and complete restriction of the six D.O.F.
4. Design of machine structure. Define the rigid mold carrier, mold support frame, ejector pins, and rigidity on the support surface; consider the effects of external forces (e.g., gravity), and available operating space.
5. Cavity design. Define cavity orientation, wall thicknesses, cavity spacing or interconnection, cavity tightness and sealing, projected surface, and cross-section design.
6. Runner-system design. Define the feeding ducts, the amount of material required per cycle, the injection or casting speed, runners, sprue, gates, cavity-nesting, reservoirs, reverberators, and computational simulations.
7. Sequential process design. Define the release agent and the surfaces with release angles; define the production cycle times, design the Process Flow Diagram (P.F.D.), SMED changes, process parameter variation, and tolerance stack-up analysis.
8. Thermodynamic process design. This includes the percentage of contraction or expansion, in-mold rheology, surface coatings, and hot zones required for special thermoregulation, melt temperature, and mold temperature [49].
9. Design considering the manufacturing process. Especially for additive manufacturing, the definition of mold impression orientation, placement of support and filler material and its interference with critical zones, the machine’s capacity where the mold will be manufactured, the cross-section of internal ducts [50].
10. Accessories and various designs. Define spare parts and useful life, a preventive and corrective maintenance plan, mounting or transportation accessories, soft tooling release documentation, safety equipment for operability, and external environment factors [51].

## 4. Conclusions and Future Work

This study provided evidence that supports the employment of RTV and SWCNT for manufacturing E-skin micropatterns. The findings presented here provide a starting point for further examination of the application of the prototypes developed with the manufacturing methodology described. The conclusions are summarized as follows:

1. Fabricating multilayer materials such as artificial electronic skin via printed soft tooling by additive manufacturing is feasible for materials with properties similar to RTV-2 and SWCNT-based nanocomposites.
2. Using RTV-2 material casting into the mold cavities, we showcased the application of soft tooling in the development and prototyping stages with very promising scalability and a low production cost approach.
3. An Ogden–Roxburgh model was successfully implemented to analyze the energy dissipation of flexible materials with values greater than R^2^ > 0.86.
4. We developed a set of guidelines for the AM-based soft tooling of e-skin.
5. We verified the electrical path of the SWCNTs’ micropattern geometries with advantages that included low volumetric and surface resistivity.
6. Future work will focus on testing the assembly of the micropatterned layers and the ability to generate capacitive phenomena to detect the deformation of the electrodes. Furthermore, the application of the design guidelines and the effect on the performance of the device remains to be developed in the future.

## Figures and Tables

**Table 1:** Physical and mechanical properties of the elastomeric matrix base material.

Property	ELASTOSIL^®^ 7600	ELASTOSIL^®^ 7683
Density	0.99 g/cm^3^ (A)	0.99 g/cm^3^ (A)
	1.05 g/cm^3^ (B)	1.05 g/cm^3^ (B)
Viscosity	4000 mPa·s (A)	1400 mPa·s (A)
	2000 mPa·s (B)	4000 mPa·s (B)
Pot life	27 min	40 min
Color	Translucent	Translucent
Hardness (Shore 00)	26	25
Elongation at break	600%	500%

**Table 2:** Nanocomposite 3,4,5 wt.% Tuball™ Matrix 601 sample preparation (120 g).

Component	Sample I Weight, g (3 wt.%)	Sample II Weight, g (4 wt.%)	Sample III Weight, g (5 wt.%)
SWCNTs Tuball™ Matrix 601	3.6	4.8	5.0
RTV-2 part A	58.2	57.6	57.0
RTV-2 part B	58.2	57.6	57.0

**Table 3:** Independent samples Kruskal–Wallis test summary.

**Group**	***N***	**Median**	**Mean Rank**		**Z-Value**
3 wt.%	12	8.0	6.3		−4.8
4 wt.%	12	11.5	19.1		0.23
5 wt.%	12	14.5	29.8		4.56
Total *N*	36	-	18.5		-
**Method**			**DF**	**K-Value**	***p*-Value**
Not adjusted for ties			2	29.27	0.00000044
Adjusted for ties			2	29.65	0.00000036

**Table 4:** Pairwise comparison results using Dunn’s nonparametric post-hoc test.

Samples	Test Statistic K	Std. Error	Std. Test Statistic	*p*-Value	*p*-Value *
3 wt.%–4 wt.%	−12.500	4.274	−2.925	0.003	0.010
3 wt.%–5 wt.%	−23.250	4.274	−5.440	<0.001	0.000
4 wt.%–5 wt.%	−10.750	4.274	−2.515	0.012	0.036

* Significance values were adjusted by the Bonferroni correction for multiple tests.

**Table 5:** Summary of constitutive constants provided by the calibration process numerical solution-based solvers for Ogden–Roxburgh model (*N* = 3) via Ansys (right) and Abaqus (left) solvers.

Property	ELASTOSIL^®^ 7600		ELASTOSIL^®^ 7683	
$\mu_{1}$ [MPa]	−0.023	0.024	1.65 × 10^−4^	0.068
$\mu_{2}$ [MPa]	−0.010	0.049	0.013	0.021
$\mu_{3}$ [MPa]	0.079	0.061	3.74 × 10^−4^	0.017
$\alpha_{1}$[–]	1.396	0.226	1.733	0.271
$\alpha_{2}$[–]	1.322	0.266	0.571	0.345
$\alpha_{3}$[–]	−2.641	0.267	−2.051	0.372
r [MPa]	1.589	1.581	1.374	1.383
m [MPa]	0.120	0.119	0.124	0.120
β [MPa]	3.36 × 10^−11^	2.27 × 10^−4^	8.74 × 10^−5^	1.67 × 10^−4^

**Table 6:** Summary results of coefficient of determination (R^2^) for Ogden–Roxburgh model using Ansys (right) and Abaqus (left) solvers and Nelder–Mead algorithm.

*N*	ELASTOSIL^®^ 7600		ELASTOSIL^®^ 7683	
3	0.865	0.871	0.921	0.918
4	0.883	0.889	0.922	0.912
5	0.894	0.897	0.916	0.921
6	0.865	0.871	0.921	0.918

## Data Availability

The data presented in this study are openly available in FigShare at https://doi.org/10.6084/m9.figshare.c.5764694.v1.
